# Aptamer-Modified Magnetic Nanoparticles as Targeted Drug Delivery Systems for Hepatocellular Carcinoma

**DOI:** 10.3390/pharmaceutics17101292

**Published:** 2025-10-02

**Authors:** Alexandra Pusta, Mihaela Tertis, Bianca Ciocan, Rodica Turcu, Izabell Crăciunescu, Victor C. Diculescu, George E. Stan, Stefan Bulat, Alina Porfire, Andreea-Elena Petru, Ionel Fizeșan, Simona Mirel, Cecilia Cristea

**Affiliations:** 1 Department of Analytical Chemistry and Instrumental Analysis, Iuliu Hațieganu University of Medicine and Pharmacy, 4 Pasteur Street, 400349 Cluj-Napoca, Romania; alexandra.pusta@umfcluj.ro (A.P.); ccristea@umfcluj.ro (C.C.); 2 Department of Medical Devices, Iuliu Hațieganu University of Medicine and Pharmacy, 4 Pasteur Street, 400349 Cluj-Napoca, Romania; smirel@umfcluj.ro; 3 National Institute for Research and Development of Isotopic and Molecular Technologies, 67–103 Donat Street, 400293 Cluj-Napoca, Romania; rodica.turcu@itim-cj.ro (R.T.); izabell.craciunescu@itim-cj.ro (I.C.); 4 National Institute of Materials Physics, 405A Atomiştilor Street, 077125 Măgurele, Romania; victor.diculescu@infim.ro (V.C.D.); george_stan@infim.ro (G.E.S.); stefan.bulat@infim.ro (S.B.); 5 Department of Pharmaceutical Technology and Biopharmaceutics, Iuliu Hațieganu University of Medicine and Pharmacy, 41 Victor Babeş Street, 400012 Cluj-Napoca, Romania; aporfire@umfcluj.ro; 6 Department of Toxicology, Faculty of Pharmacy, Iuliu Hațieganu University of Medicine and Pharmacy, 8 Victor Babeş, Street, 400012 Cluj-Napoca, Romania; andreea.elen.petru@elearn.umfcluj.ro (A.-E.P.); ionel.fizesan@umfcluj.ro (I.F.)

**Keywords:** aptamers, magnetic nanoparticles, sorafenib, hepatocellular carcinoma, targeted drug delivery

## Abstract

**Background**: Hepatocellular carcinoma is associated with high mortality and increasing incidence. Sorafenib, a cornerstone of therapy for advanced hepatocellular carcinoma, presents certain disadvantages, including low bioavailability and poor water solubility. This work describes a new strategy for sorafenib-targeted delivery aimed at improving treatment efficiency and reducing side effects. **Methods**: Magnetic nanoparticles coated with azelaic acid were modified with aptamer molecules that specifically recognize human liver cancer cell line HepG2, ensuring specificity for the tumor tissue. The nanoparticles were further loaded with sorafenib. The obtained drug delivery system was extensively characterized using UV-Vis spectrophotometry, transmission electron microscopy, X-ray diffraction, Fourier-transform infrared spectroscopy, X-ray photoelectron spectroscopy, and electrochemical impedance spectroscopy. **Results**: The drug delivery system demonstrated a higher release of sorafenib at acidic pH compared to pH 7.4. The cell internalization of the bare and aptamer-modified magnetic nanoparticles was assessed in HepG2 and human normal foreskin fibroblasts BJ cell lines, demonstrating that the aptamer significantly enhances internalization in tumor cells, while having no impact on healthy cells. **Conclusions**: The sorafenib-modified nanoparticles exhibited excellent cytocompatibility with BJ cells across all tested concentrations, while showing cytotoxicity towards HepG2 cells at higher concentrations, confirming the selectivity of the system.

## 1. Introduction

Hepatocellular carcinoma (HCC) is the most common type of liver cancer [1] and an important cause of cancer-related mortality worldwide [2]. Its increasing incidence makes HCC a prevalent public health concern. For this reason, multiple treatment strategies have been developed for HCC, ranging from liver transplantation to systemic therapy [3].

Sorafenib (SOR), a multi-kinase inhibitor drug, is one of the first-line options for the treatment of advanced HCC [1]. Despite increasing overall survival in patients with HCC, SOR has certain drawbacks such as poor water solubility [4], low bio-availability, systemic side effects [1], and reduced efficiency in certain patients [5].

Recent advances in nanotechnology offer promising strategies to overcome these drawbacks by incorporating SOR into nano drug delivery systems. These systems are based on a variety of structures such as polymeric nanoparticles [6], dendritic structures [7], liposomes [8,9], silica nanoparticles [10,11], carbon materials [12,13], or magnetic nanoparticles (MNPs) [14,15,16,17,18,19,20].

MNPs are promising candidates for drug delivery due to their high drug-loading capacity, superparamagnetism, low-cost production, and ease of modification with various chemical groups [21].

Biomedical nanomagnetics, defined as the application of MNP-based technologies in medicine, is a multi-disciplinary field that brings together expertise from medicine, materials science, and engineering. The use of existing chemotherapy drugs or genetic material conjugated to functionalized magnetic carriers enables site-specific and externally triggered drug delivery, thereby reducing the required therapeutic dose and minimizing associated adverse effects. Among candidates for magnetic nanomaterials, iron oxide, particularly magnetite nanoparticles, were proven to be the least toxic [22], making it the most promising material for medical applications. In magnetic drug targeting, drug-loaded magnetic carriers can be introduced into the bloodstream and directed by an external magnetic field to a specific body region in which they can be guided and concentrated. Significant research efforts are dedicated to suitable surface modifications of MNPs to improve colloidal stability, prevent aggregation, ensure non-toxic state in physiological conditions, and introduce functional groups for the binding of application-specific target molecules, such as antibodies, peptides, or aptamers.

Aptamers are single-stranded DNA or RNA sequences that present marked specificity towards a desired target [23]. Also named “chemical antibodies”, aptamers have high stability and specificity, long shelf-lives, low batch-to-batch variability, smaller sizes, and lower immunogenicity [23,24,25] compared to antibodies.

Several aptamer-guided drug delivery approaches for SOR have been reported in the literature. Ali et al. reported polymeric microcapsules loaded with SOR and functionalized with an anti-ErbB3 RNA aptamer. The presence of the aptamer significantly reduced the systemic side effects of SOR in mice [26]. Another approach involved the development of hollow mesoporous MnO_2_ nanoparticles functionalized with an aptamer specific to glypican-3. The obtained nanoparticles were used for theranostic applications, showing higher tumor inhibition compared to non-functionalized nanoparticles [27]. Co-encapsulation of SOR and CRISPR/Cas9 in mesoporous silica nanoparticles covered with aptamer was reported by Zhang et al. The complex displayed high anti-tumor efficiency and limited side effects [28].

In this work, the combined use of MNPs and the TLS11a aptamer for the development of SOR drug delivery systems is reported for the first time. MNPs coated with azelaic acid (MNP@AZA) were obtained and characterized. The MNP@AZA were further functionalized with the TLS11a aptamer, specific for HCC cells, thereby enhancing the selectivity of the drug delivery system toward human liver cancer cells (HepG2). The obtained aptamer-functionalized MNP@AZA (MNP_Apt), were then loaded with SOR to obtain MNP_Apt_SOR. The resulting drug delivery systems were extensively characterized, using a variety of imaging and spectroscopy techniques to confirm the success of each functionalization step. The internalization of the drug delivery system in HepG2 cells and human normal foreskin fibroblasts (BJ) was also evaluated, together with the cytotoxicity of the system in these cells.

## 2. Materials and Methods

### 2.1. Materials

#### 2.1.1. Chemicals and Reagents

Aptamer TLS11a [29] (K_d_ = 4.51 ± 0.39 nM) RP-HPLC purity was purchased from Eurogentec (Seraing, Belgium). The aptamer sequence was the following: 5′-ACA-GCA-TCC-CCA-TGT-GAA-CAA-TCG-CAT-TGT-GAT-TGT-TAC-GGT-TTC-CGC-CTC-ATG-GAC-GTG-CTG-3′ and the 5′ end was modified with an amino group and a C6 linker. Sorafenib tosylate (SOR) was purchased from Sigma-Aldrich, USA.

All other chemicals, purchased from Sigma-Aldrich, Merck Chemicals, and Honeywell Fluka, were of analytical grade and were used without additional purification. All solutions were prepared in nuclease-free water (Invitrogen, Waltham, MA, USA), unless stated otherwise.

#### 2.1.2. Cell Cultures

The HepG2 and BJ cell lines were purchased from the American Type Culture Collection (ATCC, Manassas, VA, USA) with product codes ATCC© HB-8065 and ATCC© CRL-2522™, respectively. HepG2 and BJ were maintained in Eagle’s Minimum Essential Medium (EMEM, Gibco, Paisley, UK) with high glucose (5 g/L) and in Dulbecco’s Modified Eagle Medium (DMEM, Gibco, Paisley, UK) with low glucose (1 g/L), respectively. Basal media were supplemented with 10% Fetal Bovine Serum (FBS, Sigma Aldrich, Steinheim, Germany). Both cell types were cultured at 37 °C in a humidified incubator with 5% CO_2_, and the cellular media was refreshed every 48 h. Cells were sub-cultured or harvested for experiments when they reached a confluency of 70–90%.

### 2.2. Methods

#### 2.2.1. Aptamer Solution Preparation

The aptamer stock solution was prepared in TRIS buffer pH 7.2 (0.01 M 2-Amino-2-(hydroxymethyl)-1,3-propanediol, 0.1 M NaCl, 0.1 M KCl and 0.01 M MgCl_2_). The stock solution was aliquoted and stored at –20 °C until use. Before aptamer functionalization, the aptamer stock solution was diluted with the appropriate amount of TRIS buffer and subjected to thermal denaturation by heating at 95 °C for 5 min in a Thermomixer (Eppendorf, Germany), followed by rapid cooling at –20 °C for 30 s. The aptamer dilutions were freshly prepared daily prior to use.

#### 2.2.2. Magnetic Nanoparticle Synthesis

MNPs were obtained using a previously described method [30]. Briefly, MNPs were obtained via the thermal decomposition of iron(III) acetylacetonate Fe(acac)_3_ in benzyl ether, in the presence of oleic acid and oleylamine. The mixture was subsequently heated at 150 °C and 200 °C for 1 h each, followed by refluxing at 293 °C for 30 min. The resulting mixture was cooled to room temperature. This initial synthesis step resulted in the formation of hydrophobic MNPs, coated with oleic acid.

Next, MNP@AZA were obtained by dispersing the hydrophobic MNPs in ethyl acetate–acetonitrile (1:1), followed by the addition of an oxidant (aqueous NaIO_4_ solution). This process led to the oxidative scission of the oleic acid shell and its transformation in AZA. The mixture was allowed to react for 4 h, after which it separated into an aqueous and an organic layer. The resulting MNP@AZA nanoparticles were separated from the aqueous layer using a magnet.

#### 2.2.3. Aptamer Functionalization

Aptamer TLS11a was used for the functionalization of the MNPs via amide-bond formation. The process was assisted by the use of NHS/EDC (N-hydroxysuccinimide/N-ethyl-N-(3-(dimethylamino)propyl) carbodiimide) chemistry and was divided in two distinct steps:

(i) First, the carboxyl groups were activated using a mixture of 2 mM NHS and 4 mM EDC prepared in nuclease-free water. A 5 mg/mL suspension of MNPs (3.6 mg Fe/mL) was prepared in the NHS/EDC mixture and allowed to react for 1 h under continuous shaking on a HulaMixer using the following shaking conditions: orbital shaking 3 r/min (60 s), reciprocal shaking 1° (10 s), and vibration movement 1° (5 s). Next, the MNPs were separated from the activation mixture using a magnetic stand and were washed three times with 2 mL of TRIS buffer to remove the activation mixture.

(ii) In the second functionalization step, the activated MNPs were re-suspended in a 2 μM aptamer solution prepared in TRIS and allowed to react under continuous shaking on a HulaMixer to obtain the aptamer functionalized MNPs (MNP_Apt). The MNP_Apt were separated from the supernatant using a magnetic stand and washed three times with TRIS to remove the unbound aptamer. The aptamer containing supernatant was analyzed using UV-Vis spectrophotometry before and after MNP incubation to confirm the aptamer immobilization.

#### 2.2.4. SOR Loading and Release

Next, a 2 mg/mL SOR solution was prepared in 96% ethanol and incubated with the MNP_Apt for 24 h to obtain the SOR-loaded MNPs (MNP_Apt_SOR). The SOR-containing supernatant was analyzed using UV-Vis spectrophotometry before and after MNP_Apt incubation to quantify the amount of SOR loaded onto the functionalized MNPs. The loading capacity (LC) and encapsulation efficiency (EE) were calculated using Equations (1) and (2):

EE (%) = (V × C_i_ − V × C_f_)/(V × C_f_) × 100 (1)

LC (%) = (V × C_i_ − V × C_f_)/m_NP_ × 100 (2)

where V—the volume of the loading media (2 mL), C_i_—initial concentration of SOR in the loading media, C_f_—final concentration of SOR in the loading media, after loading, m_NP_—mass of the loaded MNPs.

The C_i_ and C_f_ parameters were determined by UV-Vis spectrophotometry using a calibration curve built for SOR in 96% ethanol.

To study the release of SOR, the MNP_Apt_SOR were incubated in phosphate-buffer saline (PBS) at pH 5.5 and 7.4, respectively, under continuous shaking on a HulaMixer. 500 μL of release media were sampled at 15 min intervals during the first hour, 1 h intervals over the following 5 h, and after 24 h. After each sampling, the release media was supplemented with 500 μL of the appropriate buffer, to maintain a constant volume of release media. Prior to analysis, samples collected from the release study at pH 5.5 were diluted in PBS (pH 5.5), whereas those from the release study at pH 7.4 were diluted in ethanol exclusively for spectrophotometric measurements. The concentration of the obtained dilutions was determined using UV-Vis spectrophotometry. The cumulative release of SOR in the two types of release media was determined using Equation (3).

(3) $$C_{rn}(\%)=\frac{100\times (VC_{n}+V_{s}\sum C_{n-1})}{m_{load}}$$

where C*_rn_*—cumulative release rate; C*_n_*—SOR concentration in the release buffer at a given time; V—total volume of the release media; V*s*—volume of release media sampled at each time tested; C*_n_*_−1_—SOR concentrations in the release media at previous testing times; m*_load_*—amount of SOR loaded in the MNPs.

#### 2.2.5. Zeta Potential and Hydrodynamic Size

The zeta potential and particle size of the MNPs were measured using a Zetasizer Nano ZS (Malvern Panalytical, Malvern, UK), using laser Doppler electrophoresis for the zeta potential determination and dynamic light scattering for the size and polydispersity index (PDI) determination. The measurements were carried out in triplicate at 25 °C, after a 1:100 (*v*/*v*) sample dilution with ultrapure nuclease-free water.

#### 2.2.6. UV-Vis Spectrophotometry

UV-Vis spectrophotometry was performed to confirm aptamer functionalization and SOR loading. A SPECORD 250PLUS spectrophotometer (Analytik, Jena, Germany) was used together with the WinAspect PLUS software version 4.2.0.0. The spectra were recorded between 190 and 350 nm and calibration curves for SOR in ethanol, PBS pH 5.5, and PBS pH 7.4–ethanol (1:3) were constructed using solutions of increasing concentrations.

#### 2.2.7. Transmission Electron Microscopy

The transmission electron microscopy (TEM) investigations were performed on a chromatic aberration probe-corrected JEM ARM 200 F analytical electron microscope operated at 200 kV. Image processing was performed using specialized routines in Gatan Digital Micrograph software (version 2.x). Cross-section TEM specimens were prepared by polishing the samples mechanically down to ca. 30 μm, followed by ion milling in a Gatan PIPS machine at 4 kV accelerating voltage and 7° incidence angle. Low-voltage (2 kV) milling was used as the final ion polishing stage to reduce the amorphous surface layer enveloping the specimen.

#### 2.2.8. X-Ray Diffraction

X-ray diffraction (XRD) analysis was performed in Bragg–Brentano geometry using an XRDynamic 500 system (Anton Paar, Graz, Austria) with CuK_α_ radiation (λ = 1.5418 Å), Ni/C multilayer monochromator, and a one-dimensional Pixos 2000 detector. The XRD patterns were recorded over a 2θ range of 20–70°, with a step size of 0.02° and a time per step of 5.5 s. Grazing incidence XRD measurements were also attempted but were unsuccessful in detecting signals from the functionalization coatings. The crystalline coherence length, excluding the strain contribution, was estimated from the integral breadth of selected diffraction lines using the Scherrer equation. Instrumental broadening was corrected using LaB6 reference material.

#### 2.2.9. Fourier-Transform Infrared Spectroscopy

The chemical structure was analyzed by Fourier-transform infrared (FTIR) spectroscopy in attenuated total reflectance (ATR) mode using a Jasco 6800-FV-BB spectrometer (Jasco Corporation, Tokyo, Japan) equipped using an ATR PRO670H attachment featuring a bulk diamond crystal. FTIR-ATR spectra were acquired under vacuum with a resolution of 4 cm^−1^ over the range of 4000–200 cm^−1^, representing the average of 128 individual scans. This resolution allows the majority of vibrational bands relevant to the molecular structure to be resolved while providing a satisfactory signal-to-noise ratio. Under these conditions, peak positions are accurate within ±2 cm^−1^. A post-acquisition ATR penetration depth correction was applied to account for the wavelength-dependent variation in sampling depth and to ensure accurate representation of the spectral intensities. No spectral smoothing was performed.

#### 2.2.10. X-Ray Photoelectron Spectroscopy

The surface chemical composition of the MNP was analyzed by X-ray photoelectron spectroscopy (XPS) with SPECS spectrometer equipped with a dual-anode X-ray source Al/Mg, a PHOIBOS 150 2D CCD hemispherical energy analyzer, and a multi-channeltron detector. The vacuum was maintained at 1 × 10^–9^ torr. An Al Kα X-ray source (1486.6 eV) operating at 200 W was employed for the XPS investigations. The XPS survey spectra were recorded with a pass energy of 30 eV and a step size of 0.5 eV/step. High-resolution spectra for individual elements were obtained by accumulating multiple scans at a pass energy of 30 eV and a step size of 0.1 eV/step. To facilitate XPS measurements, the particle suspension was dried on an indium foil. Data analysis and curve fitting were performed using CasaXPS software (Version 2.3.26), employing a Gaussian-Lorentzian product function and a non-linear Shirley background subtraction method.

#### 2.2.11. Electrochemical Impedance Spectroscopy

Electrochemical impedance spectroscopy (EIS) was carried out as an alternative method to confirm the successful functionalization of the MNP with aptamer. The MNP_Apt suspension was drop-cast onto a carbon screen-printed electrode (Dropsens, Llanera, Spain), and an external magnetic field was used to ensure the immobilization of the MNP_Apt onto the working electrode. EIS was performed in 5 mM [Fe(CN)_6_]^4−/3−^ under the following conditions: 61 frequencies from 0.1 to 100,000 Hz; amplitude 0.01 V at open circuit potential, using an Autolab MAC80100 multichannel potentiostat/galvanostat (Metrohm AG, Barendrecht, The Netherlands), operated with the Nova 1.10.4 software.

#### 2.2.12. Cytotoxicity and Cell Internalization Studies

The cytotoxic effects of SOR and the synthesized nanomaterials were evaluated in parallel on the cancerous and normal cells by using the Alamar Blue (AB) assay as previously described [31]. The viability test was used to evaluate the metabolic capabilities of viable cells to transform resazurin to resorufin, a fluorescent compound. The cytotoxic effects were evaluated in 96-well plate format by exposing the cells for 24 h. Briefly, 5 × 10^3^ BJ cells and 1 × 10^4^ HepG2 cells were seeded in 100 μL of medium in 96-well plates and allowed to attach overnight. Cells were washed with PBS and then exposed to SOR and the synthesized nanomaterials for 24 h. The exposure dose for SOR was expressed as molar concentration, while for the nanoparticles and the modified nanoparticles, the dose was expressed as MNPs quantity/cm^2^. After the exposure, cellular media was removed, and the cells were incubated with the AB reagent for 2–3 h. The fluorescence of resorufin was measured at λ_excitation_ = 530/25 nm and λ_emission_ = 590/35 nm, using Synergy 2 Multi-Mode Microplate Reader.

#### 2.2.13. Evaluation of Cellular Uptake

The cellular uptake of the bare and MNP_Apt was evaluated after an incubation time of 24 h at concentrations ranging from 15 to 120 µg/cm^2^ by the Liebig reaction. Briefly, after exposure, cells were thoroughly washed with PBS to eliminate non-internalized and weakly bound NPs, then trypsinized, and centrifuged for 5 min at 4500× *g*. The cell pellet was further digested in 12% HCl to release Fe^3+^ ions, and the existing Fe^2+^ was oxidized by adding ammonium persulfate. The total Fe^3+^ was quantified spectrophotometrically based on the reaction with thiocyanate, which forms a red-colored complex that absorbs light at 485 nm. A calibration curve with Fe^3+^ concentrations from 5 to 140 µg/mL was used for the quantification.

#### 2.2.14. Statistical Analysis

The results obtained from the cytotoxicity and internalization studies were presented as the mean value ± standard deviation (SD) of 3 biological replicates (each one including 5 technical replicates). Data sets were analyzed using One-way or Two-way analysis of variance (ANOVA) with a post hoc Dunn’s Multiple Comparison Test2-, with differences considered statistically significant at *p* < 0.05. Graphical representations and statistical analysis were performed using the SigmaPlot 11.0 computer software (Systat, Software Inc., Chicago, IL, USA).

## 3. Results

### 3.1. MNP Synthesis

The main results regarding the synthesis and characterization of MNP@AZA were reported elsewhere [30]. Briefly, MNP@AZA were obtained via a simple, eco-friendly method using sodium metaperiodate (NaIO_4_) which enabled their transfer to a hydrophilic medium via oxidative cleavage of oleic acid. This process preserved their morphostructural and magnetic properties, yielding stable colloidal solutions. The resulting thin azelaic acid coating prevented aggregation and allowed control over site occupation in the spinel structure, achieving high saturation magnetization (100 emu/g for MNP@AZA).

**Figure 1:**
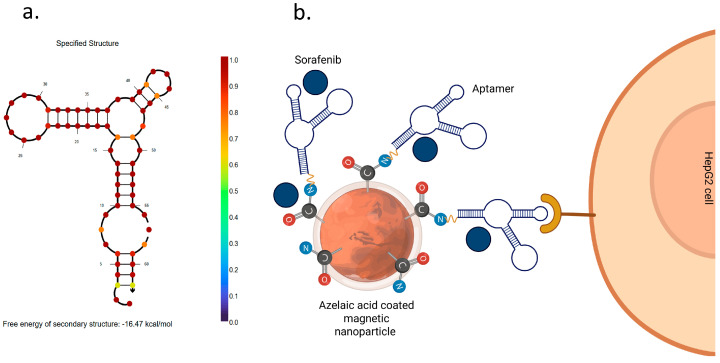
(**a**) Simulation of the secondary structure of the TLS11a aptamer performed by free-energy minimization algorithm using NUPACK web application at www.nupack.org. (accessed on 15 September 2025) (**b**) Schematic representation of the modified MNPs. Created using Biorender.com.

The focus of this work was the development and characterization of MNPs modified with the TLS11a aptamer (Figure 1) and loaded with SOR. The secondary structure of the aptamer is represented in Figure 1a, while Figure 1b provides a schematic representation of the modified MNPs and the aptamer binding mechanism.

### 3.2. Optimization of Aptamer Functionalization and SOR Loading

The following parameters were optimized in the design of the MNP_Apt_SOR: (1) aptamer incubation time; (2) aptamer incubation temperature; (3) SOR loading solution concentration; and (4) loading time. For SOR loading, the EE was calculated for each case. The data is summarized in Table 1, with the optimal parameters highlighted in bold.

For aptamer incubation, the supernatant absorbance was measured at 260 nm using UV-Vis spectrophotometry. The decrease in absorbance was attributed to the aptamer binding with the MNP@AZA. As seen in Table 1, increasing the incubation time from 30 min to 1 h led to an approximately two-fold decrease in absorbance, while an incubation period of 2 h produced no additional changes. A further increase to 24 h resulted in an additional two-fold decrease, while an incubation period of 48 h did not produce significant changes. For practical reasons, an 11.1% reduction in absorbance was deemed sufficient, so 1 h was chosen as the optimal incubation time. Moreover, to prevent conformational changes in the structure of the aptamer, the optimal incubation time was not extended to 24 h, despite the further decrease in absorbance.

**Figure 2:**
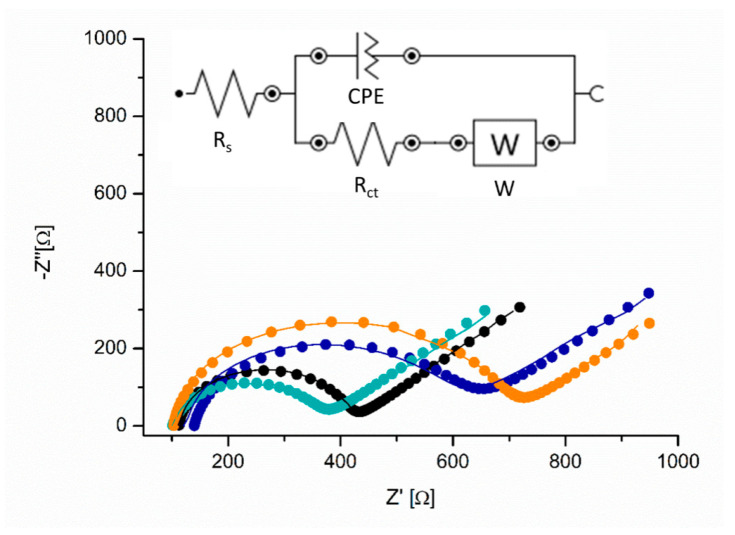
EIS spectra of the steps in the aptamer-functionalization process obtained in 5 mM [Fe(CN)_6_]^4−/3−^: bare carbon electrode (black), after MNP@AZA deposition (blue), after activation of the carboxyl groups (teal), and after aptamer incubation for 1 h (orange). Inset: equivalent circuit identified using the fitting option in Nova 1.10.4 software.

The aptamer incubation time was also optimized using EIS and the resistance to charge transfer (R_ct_) was registered after each modification step. The MNP@AZA were drop-cast on the C-SPE and an increase in the R_ct_ was recorded in 5 mM [Fe(CN)_6_]^4−/3−^, from 300 Ω to 472 Ω, confirming the presence of MNPs on the surface and their lower conductivity compared to the carbon surface. The results are presented in Table 2 and Figure 2. A decrease in the R_ct_ from 472 Ω to 259 Ω was observed after the activation of the MNP, indicating facilitated electron transfer at the surface. After aptamer immobilization, the R_ct_ increased proportionally with the aptamer incubation time, from 30 min to 48 h. This can be explained by the negative charge of the aptamer molecules, which repel the negatively charged [Fe(CN)_6_]^4−/3−^, as well as by their large size, which hinders the access of the redox probe to the electrode surface.

The Nova 1.10.4 software was used to model the processes occurring at the working electrode before and after each functionalization step. The identified equivalent circuit, [R_s_(CPE[R_ct_W])] (see the circuit as inset in Figure 2), remained consistent across all steps, incorporating solution resistance (R_s_), charge transfer resistance (R_ct_), a diffusion component (W), and a constant phase element (CPE), which replaced capacitance to account for the porous/multiphase nature of the platform. The low chi-square (χ^2^) values between 0.0039 and 0.018, obtained from the Pearson statistical test, confirmed a strong correlation between the experimental data and the proposed circuit model, indicating a good fit [32]. Figure 2 shows the Nyquist plots of the EIS data for the optimized functionalization steps, together with the corresponding modeling results from the selected fitting circuit. A strong correlation was observed between the results obtained from electrochemical impedance spectroscopy and UV-Vis spectrophotometry.

Incubating the aptamer solution with the MNP@AZA at 4 °C led to minimum aptamer binding, while room temperature incubation provided satisfactory results. An increase in the temperature to 37 °C did not produce significant changes, so 25 °C was selected as the optimal temperature for incubation.

For SOR loading, increasing the concentration to 5 mg/mL led to a significant increase in EE, so 5 mg/mL was chosen. Further concentration increases did not result in any significant changes in the EE. Doubling the incubation time to 48 h led to a 41.7% EE, indicating a saturation of the loading process. Thus, 24 h was considered as the optimal incubation time.

### 3.3. Zeta Potential and Hydrodynamic Size

The measured sizes of the MNP@AZA and MNP_Apt_SOR were 381± 24.11 nm (PDI 0.65) and 93.35 ± 22.7 nm (PDI 0.535), respectively. The notable reduction in size following aptamer functionalization and SOR loading suggests a lower tendency for aggregation and an overall improvement in the colloidal stability of the nanostructures. Such a decrease may be attributed to the stabilization effect provided by surface modifications, which reduce particle–particle interactions and enhance dispersion stability.

The zeta potential measurements further confirmed the successive functionalization steps. For MNP@AZA, a zeta potential of –18.1 mV was observed, reflecting the surface charge imparted by the azelaic acid modification. Upon aptamer attachment, the potential decreased to –24.8 mV, indicating the successful grafting of the negatively charged DNA sequence to the surface of the MNPs. Interestingly, after SOR loading, the zeta potential significantly increased to +73.7 mV. While unusual, this marked shift can be explained by the orientation of SOR molecules within the nanostructure. The hydrophobic domains of SOR are likely embedded within the carrier matrix, while protonated groups (such as the pyridine moiety under the experimental conditions) are preferentially exposed at the particle surface. This orientation results in a strong net positive surface charge. The extremely positive zeta potential is also consistent with enhanced colloidal stability, as particles with charges beyond ±30 mV are generally considered highly stable and resistant to aggregation [33]. Moreover, positively charged nanoparticles have been shown to have improved tumor uptake and retention, due to favorable electrostatic interactions with negatively charged cellular membranes [34]. The conductivity of the nanoparticle suspensions was also assessed after each modification step, to complement the zeta potential data. Thus, for the MNP@AZA a value of 0.101 mS/cm was recorded, while for the MNP_Apt and MNP_Apt_SOR, the obtained values were 0.096 mS/cm and 0.019 mS/cm, respectively. The decreasing conductivity after aptamer functionalization and SOR loading supports the occurrence of surface modifications and the restructuring of ionic environments around the nanoparticles.

These combined results, decreased hydrodynamic size, a strong positive shift in zeta potential, and decreasing conductivity, collectively support the successful functionalization of the nanoparticles, effective SOR loading via ionic interactions, and enhanced colloidal stability of the MNP_Apt_SOR system.

### 3.4. SOR Loading and Release

The loading of SOR was analyzed by calculating the LC and EE using UV-Vis spectrophotometry. The obtained LC and EE values were found to be 3.48% and 44.3%, respectively. The EE value, although relatively low, is comparable to the value reported by Depalo et al. [17] in their work related to the encapsulation of SOR and MNP in lipid micelles. It is hypothesized that SOR loading was achieved primarily by ionic interaction. Although SOR has limited water solubility and only mild basicity due to the pyridine group, the encapsulation was facilitated by electrostatic interactions between SOR and the functional groups present in the carrier system and the aptamer structure (see Figure 1b for a schematic representation of the loading mechanism).

The equations of the calibration curves used to determine SOR in different media are presented in Table 3.

**Figure 3:**
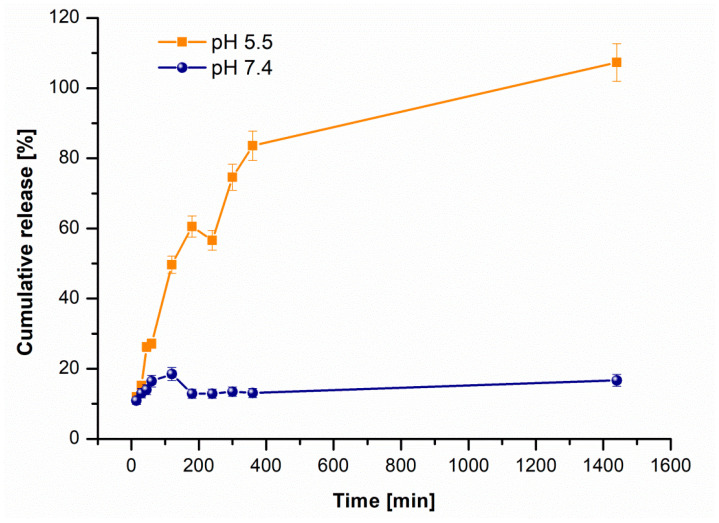
Cumulative release profiles of SOR from MNP_Apt_SOR in PBS at pH 5.5 (orange squares) and pH 7.4 (blue circles). The data represents the average of 3 determinations.

The release of SOR was evaluated in PBS at pH 5.5 and 7.4 (with triplicate measurements for each pH), and cumulative release profiles were plotted for both conditions. As shown in Figure 3, a better release profile was obtained at pH 5.5, with 97% of the loaded SOR released within the first 24 h, compared to only 16.7% at pH 7.4. The pH-dependent release profile further supports the SOR loading mechanism, a significantly enhanced release in acidic media being consistent with the disruption of ionic interactions under acidic conditions. The enhanced release at a more acidic pH offers a significant advantage for the development of anticancer therapies, as tumor tissues are known to have a slightly more acidic pH compared to healthy tissues [21]. Pharmacokinetic modeling was performed for the release profiles at pH 5.5 and 7.4. The modeling revealed that the release follows a pseudo-first order kinetics. The obtained parameters were k_1_ = 43.76 × 10^−4^, Qe = 95.06 (R^2^ = 0.954) for pH 5.5 and k_1_ = 9.21 × 10^−4^, Qe = 6.78 (R^2^ = 0.947) for pH 7.4.

Other studies have also reported on the use of MNPs for SOR delivery. A recent study developed SOR-loaded MNPs with a calculated EE value of 86% [35]. However, a cumulative release value of only 56% was reported after 24 h, compared to the 97% obtained in the present study. Another study reported the development of MNPs functionalized with polyvinyl alcohol and a double-layered hydroxide. SOR was released from the carriers over a period of 7 days, with faster release at acidic pH, but the amount released after 7 days at pH 4.8 was comparable to that released at pH 7.4 [36].

### 3.5. TEM

**Figure 4:**
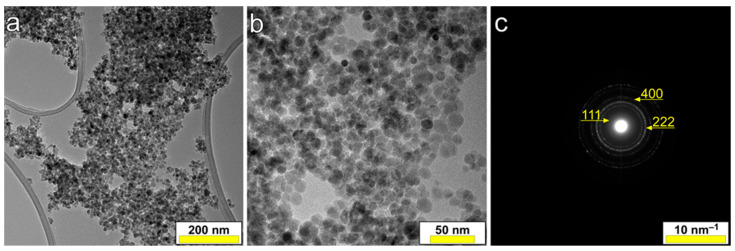
(**a**,**b**) TEM images of MNP@AZA sample collected at two different magnifications, along with a (**c**) representative SAED pattern.

TEM images were recorded for both MNP@AZA and MNP_Apt samples. Figure 4 shows the dispersion of the MNP, uniformly distributed on the surface, with quasi-spherical shape and with an average diameter of 14 nm. The corresponding selected area electron diffraction (SAED) pattern revealed the (111), (222) and (400) crystallographic planes consistent with the cubic structure of Fe_3_O_4_ (in agreement with Crystallography Open Database file no. 1513301, Fd-3m space group no. 227), thus confirming their nature. TEM and SAED analyses were also performed on MNP_Apt specimens. No remarkable morphological and structural changes could be discerned between the nanoparticles in MNP@AZA and MNP_Apt samples. This agrees with previous reports on related Fe_3_O_4_-based systems, functionalized with aptamers, which showed by TEM [37] or XRD [38] that aptamer functionalization does not alter the morphology or structure of oxide nanoparticles.

### 3.6. XRD

While conventional XRD could not provide information about the long-range order/structure of the functionalization layers due to their low thickness and density, this analysis method was used to assess any modifications in the crystalline quality of the MNPs during the different modification steps.

**Figure 5:**
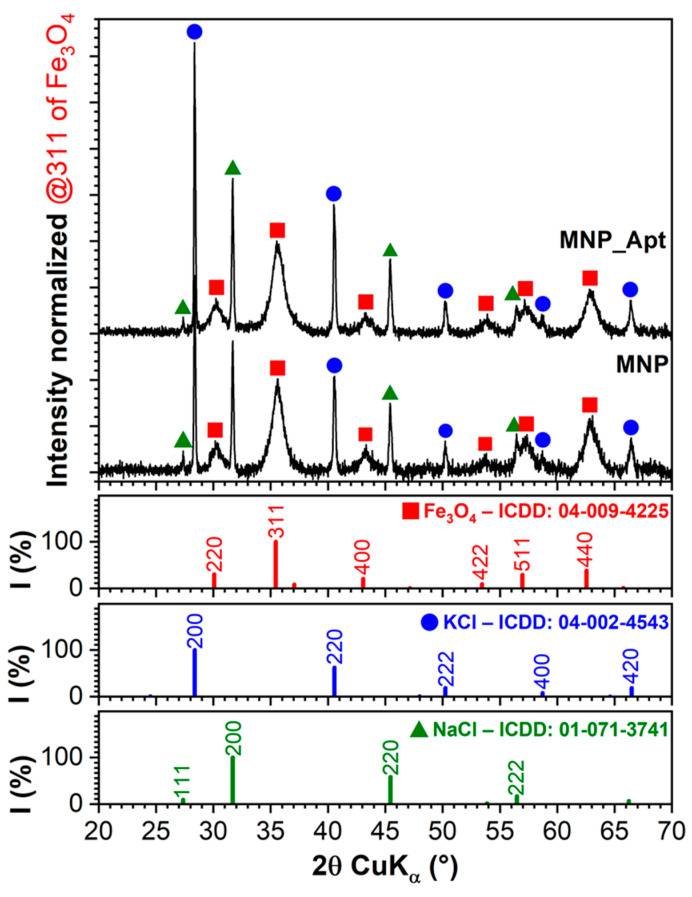
Comparative XRD patterns of the MNP@AZA and MNP_Apt samples. The intensity of the patterns was normalized relative to the maximum intensity line at 311 of the Fe_3_O_4_-like phase. The reference ICDD-PDF4 files are presented as sticks at the bottom of the graph.

The XRD patterns of the MNP@AZA and MNP_Apt samples are comparatively displayed in Figure 5. Both materials exhibit similar patterns characterized by (i) broad diffraction maxima indicative of the nanosize cubic Fe_3_O_4_-type (magnetite) phase of MNPs, and (ii) sharp peaks attributed to well-crystallized phases of KCl and NaCl, remaining after the chemical preparation and/or functionalization procedures involving TRIS buffer. The residual salts had a higher proportion in the case of the MNP_Apt sample. The crystalline coherence length (“average crystallite size”), calculated using the Scherrer equation for the maximum intensity 311 diffraction line of MNPs, was found to be 6.2 nm for the MNP@AZA sample and 5.9 nm for the MNP_Apt sample. Thus, the 14 nm nanoparticles observed in TEM (Figure 4a,b) are, on average, composed of 2–3 crystallites, as inferred from the smaller mean crystallite size (~5–6 nm) determined by XRD using the Scherrer equation. This suggests that the functionalization has marginal to no impact on the structural quality of the MNPs.

### 3.7. FTIR Spectroscopy

**Figure 6:**
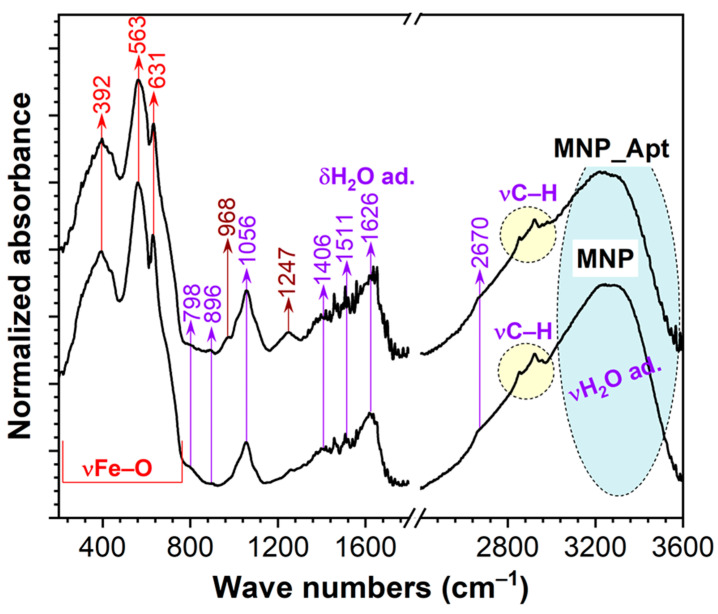
Comparative FTIR-ATR spectra of the MNP@AZA and MNP_Apt samples. The intensity of the spectra was normalized relative to the maximum intensity band originating from the MNPs.

The FTIR spectra of the MNP@AZA and MNP_Apt samples are comparatively displayed in Figure 6. The lower wavenumber region of both spectra is dominated by IR absorption stretching (ν) modes of Fe–O bonds in the magnetite-type compound, specifically 630 and 563 cm^−1^ (ν1), and 392 cm^−1^ (ν2) [39,40,41]. The presence of adsorbed water is indicated by the two broad bands at 1626 cm^−1^ and 3600–2800 cm^−1^, corresponding to the bending (δ) and stretching vibrational modes of water, respectively. The presence of residual water molecules is expected in samples produced by wet chemical procedures. Drying treatments, whether under vacuum or by heating in an oven, were not applied to avoid altering the structural chemistry of the organic moieties. Superimposed on the broad νH_2_O band are multiple stretching modes of C–H bonds [42], which could be attributed to both the functional layers and adventitious carbon resulting from exposure to air during sample storage and handling.

Both samples featured a set of common supplemental IR bands peaking at approximately 798, 896, 1045, 1406, 1511, and 2670 cm^−1^, which could be ascribed to the presence of C–H, C–Cl, C–O, N–O, C=O, and C=C bond vibrations in various residual organic moieties [43]. The aptamer functionalization of MNPs introduced two new IR bands, centered at 968 and 1247 cm^−1^ (colored in brown), which could be associated with the backbone bending and asymmetric stretching of O–P–O and (PO_2_)^−^ groups in DNA [44]. The other bands specific to DNA—e.g., C=O in guanine and thymine, C=N in adenine and guanine, C–O in deoxyribose, sugars, symmetric stretching of (PO_2_)^−^ groups [44,45]—are seemingly obscured by the more intense signals of the other organic counterparts.

### 3.8. XPS

**Figure 7:**
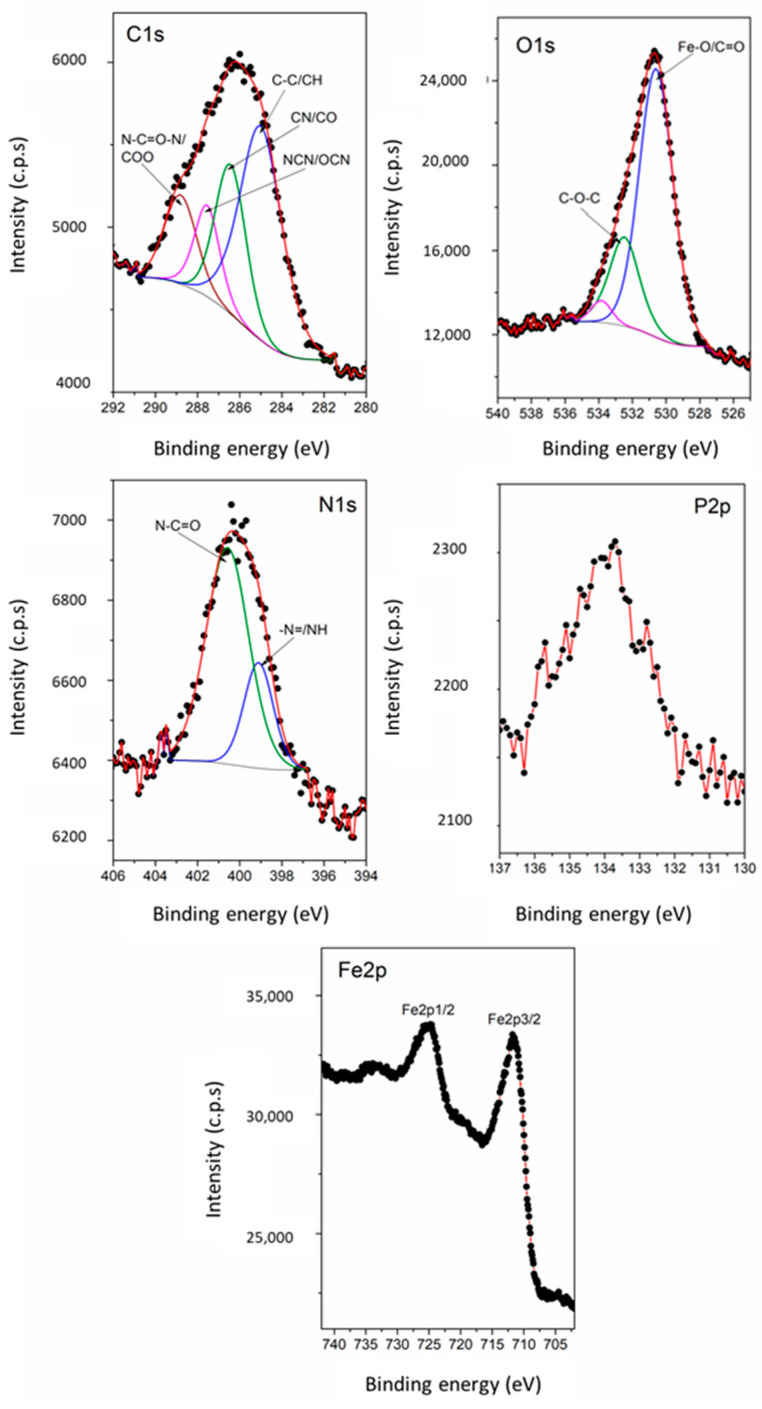
The high resolution XPS spectra of C 1s, O 1s, N 1s, P 2p, and Fe 2p core-levels of the magnetic nanoparticles functionalized with aptamer, MNP_Apt. The black dots represent the experimental XPS data.

XPS analysis was used to confirm the successful functionalization of MNPs with aptamer and their loading with SOR. Figure 7 presents the high-resolution XPS spectra of C 1s, O 1s, N 1s, P 2p, and Fe 2p core levels of the magnetic nanoparticles functionalized with aptamer, MNP_Apt. The C1s spectrum was best fitted with four components: the most intense component located at 285 eV corresponds to C–C, C–H; the peaks at 286.4 eV and 287.5 eV are attributed to CN/CO and NCN/OCN, respectively; and a higher binding energy component at 288.8 eV ascribed to N–C=O–N/COO groups [46]. In the O1s spectrum from Figure 7, two main components are observed: one assigned to Fe–O/C=O located at 530.6 eV and another to C–O–C at 532.5 eV. The N 1s spectrum from Figure 7 exhibits two components ascribed to –N=/NH at 399.1 eV and to N–C=O at 400.5 eV. The components highlighted in the XPS spectra for C 1s, O 1s, and N 1s in Figure 7 demonstrate the successful functionalization of MNPs with aptamer. Additionally, the success of this functionalization is emphasized by the P 2p and Fe 2p spectra shown in Figure 7.

**Figure 8:**
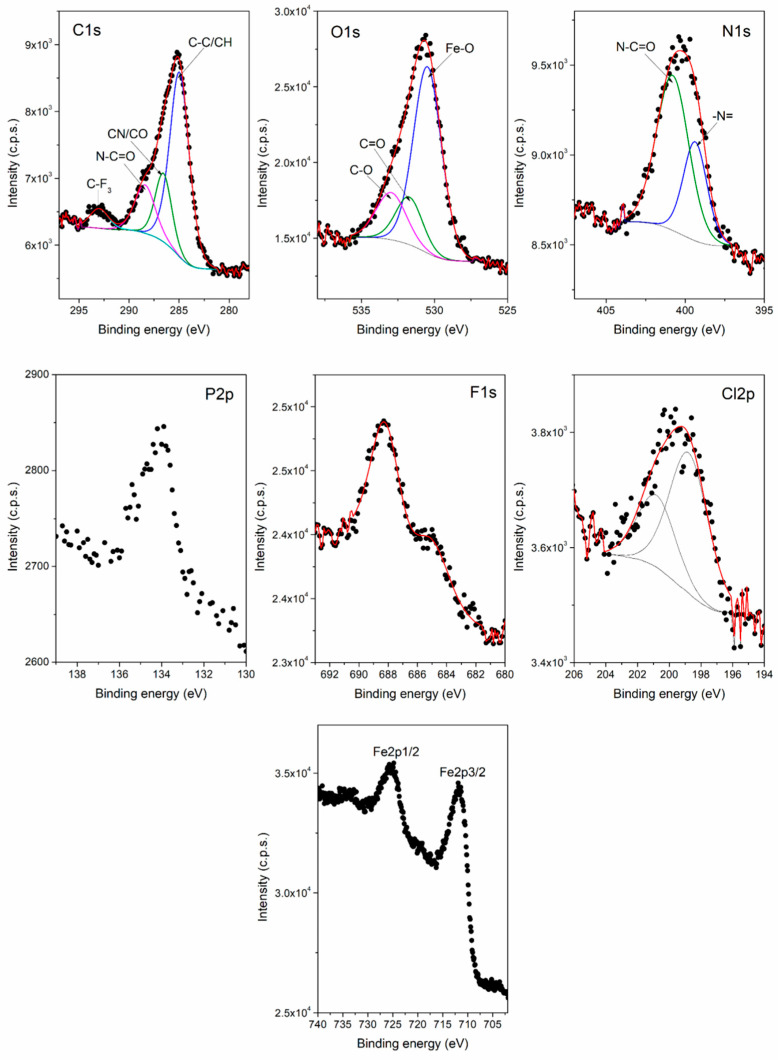
The high-resolution XPS spectra of C 1s, O 1s, N 1s, P 2p, F 1s, Cl 2p, and Fe 2p core-levels from the aptamer functionalized MNPs loaded with SOR, MNP_Apt_SOR. The black dots represent the experimental XPS data.

The high-resolution spectra of C 1s, O 1s, N 1s, P 2p, F 1s, Cl 2p and Fe 2p core-levels from MNP_Apt_SOR are shown in Figure 8. The components highlighted by the deconvolution of C 1s, O 1s, N 1s spectra, as well as P 2p, F 1s, Cl 2p spectra, are characteristic of the molecular structure of SOR. The C 1s spectrum from Figure 8 contains four components ascribed to C–C/CH at 285 eV, CN/CO at 286.6 eV, N–C=O at 288.1 eV, and C–F_3_ at 293 eV. The O 1s spectrum exhibited three components assigned to Fe–O at 530.5 eV, C=O at 531.7 eV, and C–O at 532.9 eV.

The N 1s spectrum showed two components corresponding to –N= at 399.3 eV and N–C=O at 400.8 eV. The atomic concentrations (at.%) were calculated from the XPS spectra for MNP_Apt_SOR, and the following values were obtained: C—33.6 at. %, O—47.1 at.%, N—5.3 at.%, P—0.7 at.%, F—3.4 at.%, Cl—1.2 at.%, and Fe 8.7 at.%. These results indicate a F/Cl atomic ratio of 2.8, which closely matches the characteristic value 3 for the SOR molecular structure. Thereby, the XPS investigations confirmed the successful SOR loading of the aptamer functionalized magnetic nanoparticles.

### 3.9. In Vitro Cytotoxicity and Cellular Uptake Evaluation

**Figure 9:**
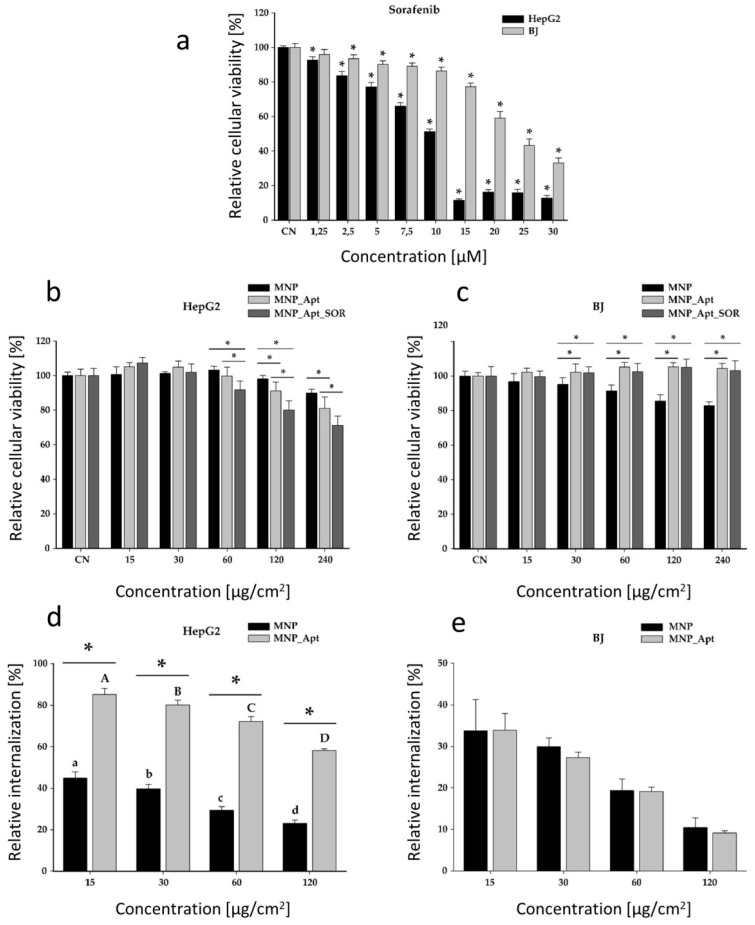
Cytotoxicity evaluation of SOR, MNP, MNP_Apt, and MNP_Apt_SOR on HepG2 and BJ cells (**a**–**c**). The results were expressed as mean ± SD (n = 3). Asterisks (*) indicate significant differences (*p* < 0.05) compared to NC. Internalization study of MNPs and MNP_Apt in HepG2 and BJ cells (**d**,**e**). Different letters indicate significant differences (ANOVA + Holm–Sidak post hoc test at *p* < 0.05).

The cytotoxic effects of SOR were assessed on both cancerous HepG2 and healthy BJ cell lines (Figure 9a), followed by the determination of the cytotoxic effects of the obtained magnetic nanostructures on HepG2 (Figure 9b) and BJ cell lines (Figure 9c). Additionally, their internalization in both cell lines was investigated (HepG2—Figure 9d, BJ—Figure 9e).

The cytotoxic effects of SOR were evaluated on cancerous and normal cell types up to a concentration of 30 µM (Figure 9a). Starting from the lowest dose of 1.25 µM, SOR statistically reduced the cellular viability of the cancerous HepG2 cells. In contrast, in the case of normal BJ cells, a statistically significant reduction was observed from the second lowest dose of 2.5 µM. For both cell types, the cytotoxicity was dose-dependent, with a higher cytotoxicity in the case of cancerous HepG2 cells. A two-way ANOVA, with the concentration and the cellular type as variables, indicated that the cancerous cells were more susceptible to the cytotoxic effects of SOR (Figure 9a). The calculated IC50 for HepG2 and BJ cells were 9.13 µM and 23.71 µM, respectively.

The toxicity of the MNP@AZA was evaluated on both cell types up to an exposure dose of 240 µg/cm^2^. The dosimetry was expressed as quantity per surface to account for the sedimentation of the MNPs and better reflect the actual dose at which the cells are exposed. The synthesized MNPs were cytocompatible with both cell types; at the highest tested dose (240 µg/cm^2^), cell viability decreased by only 10% in HepG2 cells and 18% in BJ cells (Figure 9b,c).

The notably higher apparent cytotoxicity of bare MNPs compared to MNP-Apt-SOR likely reflects surface passivation and colloidal stabilization-conferred biocompatibility upon functionalization. Multiple studies indicate that coating iron oxide nanoparticles reduces oxidative stress and iron-ion mediated toxicity. For instance, silica shell coating was shown to diminish reactive oxygen species generation and intracellular iron release, thereby improving biocompatibility versus bare MNPs [47]. Similarly, surface functionalization with polymers such as PEG or polysaccharides enhances nanoparticle stability in biological media, reducing aggregation, sedimentation, and unwanted cellular interactions [48,49]. Moreover, the surface modification of superparamagnetic iron oxide nanoparticles (SPIONs) with poly(acrylic acid) significantly reduced oxidative stress and DNA damage in human cells compared to unmodified SPIONs [50,51]. Thus, the aptamer and sorafenib coating likely shields nanoparticle reactive surfaces, leading to lower acute toxicity compared to uncoated MNPs. Secondly, the unmodified MNPs exhibited a larger hydrodynamic size in our measurements, which can lead to faster sedimentation and higher local particle concentrations at the cell monolayer. This increased local dose can contribute to higher apparent toxicity compared to more dispersed or sterically stabilized functionalized nanoparticles. This phenomenon has been observed for iron oxide nanoparticles, where aggregation and poor colloidal stability enhanced cellular uptake and cytotoxicity relative to polymer-coated, stable particles [52].

Regarding the cellular internalization of bare MNPs, a dose-dependent decrease in the relative internalization was observed for both cell types. This decrease was most likely related to the saturation in the active internalization via endocytosis, which is the dominant cellular process involved in nanoparticle uptake [53,54]. At the lowest evaluated dose of 15 µg/cm^2^, the cellular internalization was 45% of the total exposure dose in the case of HepG2 cells, and 34% in the case of BJ cells. At the highest tested dose of 120 µg/cm^2^, the recorded relative internalization was 23% of the total exposure dose in the case of HepG2 cells, and 10% in BJ cells (Figure 9d,e). Statistical analysis of these data sets indicated that HepG2 cells internalize more NPs than normal cells. These results are most likely due to the increased metabolic activity of cancerous cells, which are associated with higher nutrient demands and endocytic potential.

Similarly to bare NPs, the cytocompatibility and cellular uptake of MNP_Apt were evaluated on both cell types. Aptamer conjugation profoundly impacted the cellular uptake in cancerous cells, enhancing the internalization by two folds at all exposure doses. By increasing the exposure dose, the effect of aptamer conjugation augmented the internalization by 1.9, 2, 2.4, and 2.5 times. In the case of normal cells, this modification had no impact on the cellular uptake, indicating the selectivity of the aptamer towards HepG2 cells (Figure 9d,e). Moreover, the increased selectivity of the MNP_Apt for the HepG2 cells was observed in the cell viability data sets. Aptamer conjugation increased the cytotoxicity at the higher doses in HepG2 cancerous cells, while reducing the toxicity toward normal BJ cells (Figure 9b,c).

The cytotoxicity of MNP_Apt_SOR was similarly evaluated on both cell lines. Compared with MNP_Apt, an additional cytotoxic effect due to SOR’s presence was observed only at the highest tested concentrations, namely 60, 120 and 240 µg/cm^2^, in HepG2 cells. This modest effect could be attributed to deficiencies in SOR release within the cellular environment. On the other hand, MNP_Apt_SOR showed no toxicity toward BJ cells across the entire tested concentration range, demonstrating its selectivity towards tumor cells, with promising results in this regard. Compared with bare MNPs, functionalization with the aptamer mitigated the unspecific cytotoxic effects of MNPs and SOR, with no decrease in the viabilities of normal cells being observed for the entire range of exposure (Figure 9c). Considering the LC of 3.48%, and the exposure conditions, a dose of 240 µg/cm^2^ MNP_Apt_SOR would be equivalent to a SOR concentration of approximately 30 µM upon full extracellular release, which is not congruent with the viability data recorded (Figure 9a). Taken together, these data indicate that the developed system is highly stable in extracellular conditions, not passively releasing SOR. Additionally, the data obtained on the BJ cells indicate that the modest anticancer effect observed for HepG2 cells can be attributed solely to the internalized nanosystem. To enhance cytotoxicity against HepG2 cells and to compensate for the reduced intracellular release of SOR, the use of higher SOR concentrations during the loading process could be considered.

## 4. Conclusions

In this work, proof-of-concept, aptamer-modified, sorafenib-loaded MNPs were developed for potential applications in the targeted treatment of hepatocellular carcinoma. The MNPs were synthesized using a thermal decomposition method and functionalized with azelaic acid. The carboxyl groups served as anchoring sites for TLS11a aptamer, which has high specificity for HepG2 human hepatocellular carcinoma cells. The aptamer-functionalized MNPs were loaded with sorafenib. The obtained drug delivery system was extensively characterized using a variety of analytical methods: UV-Vis spectrophotometry, TEM, XRD, FTIR spectroscopy, XPS, and electrochemical impedance spectroscopy. The aptamer-modified MNPs demonstrated high internalization in HepG2 cells, indicating the advantage of using aptamers as ligands for active targeting and increased specificity. Moreover, their cytotoxicity was significantly more pronounced in HepG2 cells compared to healthy BJ cells, demonstrating the importance of aptamer functionalization and the suitability of the developed systems for targeted delivery. Despite the encouraging results obtained, it is important to acknowledge certain practical limitations of the developed MNPs. Specifically, their modest therapeutic efficacy and relatively low loading capacity may restrict their immediate applicability in more demanding biomedical contexts. These challenges highlight the need for continued optimization of particle design and functionalization. Potential strategies to overcome these limitations include fine-tuning the synthesis conditions to improve uniformity and surface area, incorporating biocompatible polymers or ligands to enhance drug loading, and developing hybrid nanostructures that combine magnetic cores with porous or layered coatings. Such approaches could significantly increase the efficiency and versatility of MNP-based systems, ultimately facilitating their translation toward clinical applications.

This study is limited by its reliance on in vitro assays; in vivo validation and long-term toxicity assessments are needed to substantiate translational potential. However, the presented preliminary results highlight the promising applications of aptamer-functionalized MNPs as drug carriers for hepatocellular carcinoma treatment.

## Figures and Tables

**Table 1:** Optimization of aptamer functionalization and SOR loading.

**Aptamer functionalization**		
**Optimized Parameter**	**Conditions**	**Decrease in A (%)**
Incubation time (h)	½	5.1
	**1**	**11.1**
	2	11.8
	24	20.0
	48	24.2
Incubation temperature (°C)	4	0.83
	**25**	**11.1**
	37	13.4
**SOR incubation**		
**Optimized parameter**	**Conditions**	**EE (%)**
SOR concentration (mg/mL)	1	14.8
	**5**	**44.3**
Incubation time (h)	**24**	**44.3**
	48	41.7

**Table 2:** EIS results obtained for aptamer incubation time optimization.

Conditions	R_ct_ (Ω)
Bare electrode	300
MNP@AZA	472
MNP@AZA-activated	259
MNP@AZA_Apt ½ h	399
MNP@AZA_Apt 1 h	584
MNP@AZA_Apt 2 h	602
MNP@AZA_Apt 24 h	721

**Table 3:** UV-vis parameters for the quantification of SOR in different media.

Media	λ_max_	Calibration Curve Equation	R^2^
Ethanol 96%	266	A = 0.0707[SOR] + 0.2313	0.9834
PBS pH 5.5	262	A = 0.0212[SOR] + 0.0141	0.9894
PBS pH 7.4:ethanol (1:3)	269	A = 75.62[SOR] − 0.0019	0.9974

## Data Availability

The data set is available from the authors on request.

## Abbreviations

The following abbreviations are used in this manuscript:

BJ	Human normal foreskin fibroblasts
DMEM	Dulbecco’s Modified Eagle Medium
EDC	N-ethyl-N-(3-(dimethylamino)propyl)carbodiimide
EE	Encapsulation Efficiency
EIS	Electrochemical Impedance Spectroscopy
EMEM	Eagle’s Minimum Essential Medium
FBS	Fetal Bovine Serum
FTIR	Fourier-Transform Infrared Spectroscopy
HCC	Hepatocellular Carcinoma
LC	Loading Capacity
MNP	Magnetic Nanoparticles
MNP@AZA	Magnetic Nanoparticles modified with Azelaic Acid
MNP_Apt	Magnetic Nanoparticles modified with Azelaic Acid and Aptamer TLS11a
MNP_Apt_SOR	Magnetic Nanoparticles modified with Azelaic Acid, Aptamer TLS11a and Sorafenib
NHS	N-hydroxysuccinimide
PBS	Phosphate-Buffered Solution
PDI	Polydispersity Index
R_ct_	Resistance to Charge Transfer
SAED	Selected Area Electron Diffraction
SD	Standard Deviation
SOR	Sorafenib
SPIONs	Superparamegnetic iron oxide nanoparticles
TEM	Transmission Electron Microscopy
XPS	X-ray Photoelectron Spectroscopy
XRD	X-ray Diffraction
